# Genetic relatedness of the *Escherichia coli* fecal population and strains causing urinary tract infection in the same host

**DOI:** 10.1002/mbo3.759

**Published:** 2018-10-25

**Authors:** Maryam Bahadori, Mohammad Motamedifar, Abdollah Derakhshandeh, Roya Firouzi, Azar Motamedi Boroojeni, Mohsen Alinejad, Zahra Naziri

**Affiliations:** ^1^ Department of Pathobiology, School of Veterinary Medicine Shiraz University Shiraz Iran; ^2^ Department of Bacteriology and Virology, Shiraz Medical School, Shiraz HIV/AIDS Research Center, Institute of Health Shiraz University of Medical Sciences Shiraz Iran; ^3^ Faculty of medicine Kerman University of medical sciences Kerman Iran

**Keywords:** antibiotic resistance, *Escherichia coli*, PFGE, phylogroup, UTI, virulence genes

## Abstract

It is common knowledge that fecal microbiota is a primary source of *Escherichia coli* causing urinary tract infections (UTIs) via the fecal‐perineal‐urethral route. But, it is still unknown whether *E. coli* UTI is mainly caused by dominant fecal *E. coli* isolates (prevalence hypothesis) or the isolates that possess more virulence factors (special pathogenicity hypothesis). In the present study, the urine *E. coli* isolates of 30 women with UTI were compared with the fecal *E. coli* isolates of the same patients and healthy control individuals according to the phylogenetic group, virulence genotype, and antibiotic susceptibility pattern. The genetic relatedness of the isolates was specified and compared by pulsed‐field gel electrophoresis (PFGE). PFGE analysis showed that most patients (73.3%) had distinct urine isolates which were not similar to any of their fecal isolates. Based on the phylogenetic analysis, most of the urine and fecal isolates of healthy women were assigned to phylogenetic group B2, followed by D. The distribution of phylogenetic groups was significantly different between the urine and the fecal isolates of patients (*p* < 0.05). The prevalence of *fimH* and *ompT* among urine isolates was significantly more than that among fecal isolates. The level of multidrug resistance was higher among urine isolates. Although more in‐depth researches are required, the present study could be supported by pathogenicity hypothesis. Furthermore, concerning the antibiotic resistance pattern among uropathogenic *E. coli* should be highly considered.

## 1 INTRODUCTION

The major cause of urinary tract infections (UTIs) is *Escherichia coli* (*E. coli*), which is the most common bacteria associated with more than 80% of all such infections (Schaeffer, 2005). *Escherichia coli* strains are the normal inhabitants of the human intestine which can be commensal or pathogenic (Kaper, Nataro, & Mobley, 2004). Uropathogenic *E. coli* (UPEC) is grouped as extraintestinal pathogenic *E. coli* (ExPEC) which can also make up a part of the *E. coli* strains of intestinal microbiota (Kaper et al., 2004). ExPEC strains (including UPEC) differ from commensal strains concerning certain parameters such as virulence factors and the prevalence of phylogenetic groups (Micenková, Bosák, Vrba, Ševčíková, & Šmajs, 2016).

Phylogenetic group B2 is the most prevalent group among UPEC strains, followed by D, whereas commensal strains mostly belong to group A (Bailey, Pinyon, Anantham, & Hall, 2010b). Moreover, UPEC strains contain more such virulence factors as toxins, adhesions, protections, and iron uptake systems (Siqueira et al.., 2009). It is generally believed that UPEC strains originate from intestinal microbiota, whereas there is evidence corroborating the fact that vaginal microbiota, contaminated food, animal contact, or sexual activities can also be the origin of these infections (Bélanger et al., 2011; Foxman et al., 2002; Singer, 2015).

However, there is still considerable uncertainty with regard to the pathogenesis of UTI. Two hypotheses have been suggested for the pathogenesis of this infection: (a) *E. coli* strains causing UTI are the dominant fecal strains of the host (the prevalent hypothesis); and (b) UPEC strains usually encode more virulence factors than fecal strains (the special pathogenicity hypothesis) (Moreno et al., 2008; Srivastava, Agarwal, Srivastava, & Mishra, 2014). Multidrug resistance in UPEC is increasing worldwide, leading to UTI complications and antibiotic therapy failures. Moreover, commensal *E. coli* strains can be important reservoirs for numerous antibiotic resistance genes (Bailey, Pinyon, Anantham, & Hall, 2010a).

In the present study, via pulsed‐field gel electrophoresis (PFGE), we specified the genetic relatedness between UPEC strains and *E. coli* fecal microbiota isolated from women suffering from UTI and healthy women. The prevalence of phylogenetic groups, virulence genes, and antibiotic susceptibility was also determined and compared. Notwithstanding the myriad investigations, few studies, as far as we know, have drawn such comparison. We believe that the results of this study may improve the current knowledge of the mechanism of UTI.

## 2 MATERIALS AND METHODS

### 2.1 Sample collection

From December 2014 to June 2015, through a clean catch method, 30 midstream urine specimens were obtained from nonhospitalized women with documented *E. coli* UTI who referred to various hospitals of Kerman, Iran. Fecal samples were further collected in sterile plastic containers from the same group at the time of urine collection. The patients were married women between 20 and 70 years who had not received antimicrobial treatment within 30 days before the presentation. Fecal samples were also collected from 10 healthy women who had not had a UTI at least 1 year prior to sampling.

### 2.2 Isolation, identification, and storage of *E. coli* strains

In studies of fecal microbiota, a random sample of three *E. coli* colonies would have a 97% chance of containing one isolate of the dominant clone (Lidin‐Janson, Kaijser, Lincoln, Olling, & Wedel, 1978). Hence the fact, three colonies were randomly picked from the original plates. In order to isolate *E. coli*, samples were directly inoculated on MacConkey agar plates. After overnight incubation at 37°C, lactose‐fermenting colonies were streaked on EMB agar. Typical *E. coli* colonies (with metallic sheen color) were confirmed by biochemical tests including catalase and oxidase, IMViC, motility, sugars fermentation, l‐lysine decarboxylase and sulfide production and urea hydrolysis (Markey, Leonard, Archambault, Cullinane, & Maguire, 2013). Isolates which exhibited a biochemical profile of *E. coli* were grown in LB broth (Merck‐ Germany), and kept as stock in a 25% glycerol solution at −70°C until used.

### 2.3 DNA extraction

Fresh overnight cultures were prepared, and DNA extraction was done using the boiling method.

### 2.4 Phylogenetic analysis

The phylogenetic group distribution of the *E. coli* isolates was specified using the Clermont triplex PCR method which detects the presence or absence of three DNA markers (*chuA*,* yjaA* and DNA fragment TSPE4.C2) (Gordon, Clermont, Tolley, & Denamur, 2008).

### 2.5 Detection of virulence genes

Virulence factors were characterized by amplifying the corresponding genes (*cnf‐1*,* ompT*,* hlyD*,* papA*,* fimH,* and *malX*) by PCR. Specific primers used to amplify fragments are listed in Table 1. Multiplex PCR condition for *ompT* and *hlyD* was as follows: 10 min at 94°C, 25 cycles of 30 s at 94°C, 30 s at 63°C, 3 min at 68°C, and a final extension of 10 min at 72°C. Multiplex PCR condition for *papA*,* fimH*, and *malX* was the same except for an annealing temperature of 57°C. The PCR condition for *cnf‐1* was the same except for an annealing temperature of 60°C.

**Table 1:** Oligonucleotide primers used in the study (Rodriguez‐Siek et al., 2005)

Targeted genes	Primer sequences (5′–3′)	Amplicon size (bp)
*malX*	GGACATCCTGTTACAGCGCGCA	925
	TCGCCACCAATCACAGCCGAAC	
*cnf−1*	ATCTTATACTGGATGGGATCATCTTGG	1,105
	GCAGAACGACGTTCTTCATAAGTATC	
*ompT*	ATCTAGCCGAAGAAGGAGGC	559
	GGCCAATAAATAATTTCCCGAATC	
*hlyD*	TATTAATCTTCACAGAGGAG	930
	GGCCAATAAATAATTTCCCGAATC	
*papA*	GACGGCTGTACTGCAGGGTGTGGCG	328
	ATATCCTTTCTGCAGGGATGCAATA	
*fimH*	ATTCCTCACAATCAGCGCACTT	508
	ATCAGCAGTACAGCAAACAGGG	

### 2.6 Antibiotic susceptibility testing

The antibiotic susceptibilities of *E. coli* isolates to 12 antibiotics were determined by the disk diffusion method in accordance with the Clinical and Laboratory Standards Institute (CLSI, 2017) guidelines using commercial antimicrobial disks. The antibiotics investigated in this study included third‐generation cephalosporins (ceftazidime, cefixime, cefotaxime, and ceftriaxone), trimethoprim (trimethoprim‐sulfamethoxazole), aminoglycosides (gentamicin and amikacin), fluoroquinolons (ciprofloxacin and nalidixic acid), first‐generation cephalosporins (cephalexin), carbapenems (imipenem), and nitrofurantoin. Multidrug‐resistant (MDR) strains were defined as those resisting to three or more antimicrobial classes. For the purpose of analysis, intermediate susceptibility was considered as resistance.

### 2.7 Pulsed‐field gel electrophoresis

**Figure 1:**
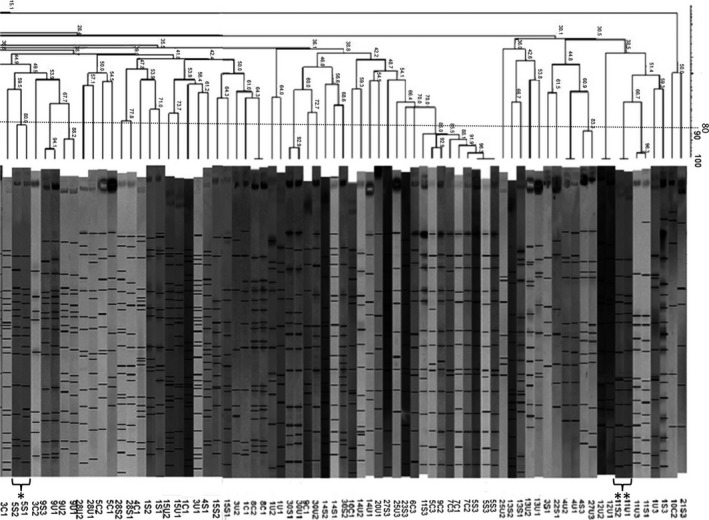
Similarity dendrogram and pulsed‐field gel electrophoresis (PFGE) profiles. C: control isolate; S: patient fecal isolate; U: urine isolate, *: two PFGE profiles with 80% similarity, **: a urine and fecal isolate from same patient with 100% similarity

Pulsed‐field gel electrophoresis was carried out on two *E. coli* isolates from each sample using *Xba*I restriction endonucleases (Thermo Scientific, Waltham, MA), as established by the Centers for Disease Control and Prevention, 2013 (using the PulseNet One‐Day [24–28 hr] Standardized Laboratory Protocol). The digested DNA was electrophoresed in the CHEF‐DR III pulsed‐field system (Bio‐Rad Laboratories, Hercules, CA) using 1% LF^TM^ agarose (X174; Amresco, Solon, OH). The profiles were analyzed using BioNumerics v7.1 fingerprinting software (Applied Maths, Sint.‐Martens‐Latem, Belgium). The cluster analysis of the Dice similarity indices using the unweighted pair‐group method with averaging (UPGMA; band tolerance: 1.5%, optimization: 1.5%) was carried out to generate a dendrogram, describing the relationship (percentage of similarity) among PFGE profiles. The isolates were considered to belong to the same PFGE cluster if their Dice similarity index was 80%, based on Tenover's criteria (Mysorekar, & Hultgren, 2006). It is shown in Figure 1 that how many band differences corresponded to <80% similarity.

### 2.8 Statistical analysis

Statistical analysis was performed using Pearson's Chi‐square test and Fisher's exact test (two‐tailed) with SPSS version 19.0; *p* values <0.05 were considered statistically significant.

## 3 RESULTS AND DISCUSSION

### 3.1 *Escherichia coli* isolation

A total of 210 *E. coli* isolates were collected, comprised of 90 urine isolates (three isolates from each urine sample), 90 patients’ fecal isolates, and 30 controls’ fecal isolates (three isolates from each fecal sample).

### 3.2 Phylogenetic analysis

The prevalence of phylogenetic groups among the isolates of three investigated groups is shown in Table 2. Phylogenetic group B2 was the most prevalent group among urine isolates followed by D, a finding in agreement with several studies which indicated that ExPEC strains belonged mostly to group B2 and D (Chakraborty et al., 2015; Nielsen, Dynesen, Larsen, & Frimodt‐Møller, 2014). Contrary to our expectations, there was a significant difference between urine and fecal isolates of the patients concerning the distribution of phylogenetic groups (*p* < 0.05). The predominant phylogenetic groups among fecal isolates obtained from patients were groups A and D. We did not expect to find out that the distribution of phylogenetic groups was similar between urine and controls’ fecal isolates. In agreement with this finding, a previous study showed that healthy women who had never previously had UTI carried fecal *E. coli* isolates closely related to the urine and fecal isolates from UTI patients (Nielsen et al., 2017). Although group B2 was the most common, followed by D among control's isolates, none of them caused infection. It is suggested that the acquisition of a potentially pathogenic phylotype is not in itself associated with UTI risk, yet further investigations are required.

**Table 2:** The number (%) of phylogenetic groups among the isolates of three studied groups

Phylogenetic groups	Source of isolates			Total[Link] (%)
	Urine (%)	Patients’ fecal (%)	Controls’ fecal (%)	
A	13 (14.4)	29 (32.2	5 (16.7)	47 (22.3)
B1	6 (6.7)	11 (12.2)	3 (10.0)	20 (9.5)
B2	53 (58.9)	22 (24.4)	12 (40.0)	87 (41.4)
D	18 (20.0)	28 (31.1)	10 (33.3)	56 (26.9)

Three isolates were picked from each urine/fecal sample.

Some studies compared the fecal microbiota of the healthy individuals and UTI patients (Moreno et al., 2009; Nielsen et al., 2014). Moreno et al. showed that the fecal microbiota of UTI patients had a significantly higher proportion of B2 isolates (Moreno et al., 2008, 2009 ). Nielsen et al. (2014) indicated similar trends with regard to the phylogenetic distribution of fecal *E. coli* from patients and healthy controls. The number of fecal isolates examined in the previous studies was more than that in the current study, and both dominant and nondominant fecal isolates were characterized (Moreno et al., 2009; Nielsen et al., 2014). Moreno et al. (2009) examined the fecal isolates of healthy individuals with no UTI 2 months prior to the time of sampling. The fecal microbiota of healthy women who never had a UTI was examined by Nielsen et al. (2014), whereas in the present study, healthy controls did not have UTI at least a year before the study. These differences in part clarify the discrepancies between the studies.

### 3.3 Prevalence of virulence genes

The frequencies of virulence genes among the isolates of four phylogenetic groups belonging to each collection are shown in Table 3. UPEC strains produce different types of adhesions, including type 1 fimbriae and p fimbriae, which are essential for the attachment and colonization of UPEC in the urinary tract (Tarchouna, Ferjani, Ben‐Selma, & Boukadida, 2013). The adhesive component of type‐1 fimbriae is encoded by the *fimH* gene (Ulett et al., 2013). Our findings demonstrated that *fimH* was the most frequent of virulence genes that were significantly more prevalent among the urine isolates (*p* < 0.05), in line with the results of several other studies indicating that *fimH* is the most prevalent virulence factor between UPEC isolates (Qin et al., 2013; Zhao et al., 2009). The major subunit of p fimbriae is encoded by *papA* (Tarchouna et al., 2013). We observed no statistically significant difference among the three groups concerning the prevalence of *papA*. In contrast, Johnson, Owens, Gajewski, & Kuskowski, 2005 reported that the prevalence of adhesion genes is different between commensal *E. coli* and UPEC strains.

**Table 3:** The phylogenetic distribution of virulence genes among all studied isolates

	Source of isolates															Total *N*. (%)
	Urine *N*. (%)					Patients’ fecal *N*. (%)				Controls’ fecal *N*. (%)						
	A	B1	B2	D	Total	A	B1	B2	D	Total	A	B1	B2	D	Total	
Adhesion genes																
*fimH*	10 (11.0)	3 (3.3)	46 (51.1)	11 (12.2)	70 (77.7)	17 (18.8)	2 (2.2)	17 (18.8)	20 (22.2)	56 (62.2)	1 (3.33)	0 (0.0)	12 (40.0)	5 (16.6)	18 (60.0)	144 (68.5)
*papA*	2 (2.2)	1 (1.1)	16 (17.7)	3 (3.3)	22 (24.4)	7 (7.7)	0 (0.0)	2 (2.2)	13 (14.4)	22 (24.4)	2 (6.6)	0 (1.1)	2 (6.6)	4 (13.3)	8 (26.7)	52 (24.4)
Toxin genes																
*cnf1*	0 (0.0)	0 (0.0)	3 (3.3)	0 (0.0)	3 (3.3)	0 (0.0)	1 (1.1)	1 (1.1)	0 (0.0)	2 (2.2)	0 (0.0)	0 (0.0)	0 (0.0)	0 (0.0)	0 (0.0)	5 (2.3)
*hlyD*	0 (0.0)	1 (1.1)	14 (15.5)	2 (2.2)	17 (18.8)	1 (1.1)	2 (2.2)	3 (3.3)	6 (6.6)	12 (13.3)	3 (10.0)	0 (0.0)	5 (16.6)	2 (6.6)	10 (33.3)	39 (18.5)
Cell protection genes																
*ompT*	5 (5.5)	2 (2.2)	25 (27.7)	5 (5.5)	37 (41.1)	6 (6.6)	0 (0.0)	8 (8.8)	2 (2.2)	16 (17.7)	2 (6.6)	0 (0.0)	1 (3.3)	3 (10.0)	6 (20.0)	59 (28.0)
Other genes																
*malX*	2 (2.2)	1 (1.1)	21 (23.3)	11 (12.2)	35 (38.8)	1 (1.1)	1 (1.1)	5 (5.5)	18 (20.0)	25 (27.7)	0 (0.0)	0 (0.0)	1 (3.3)	5 (16.6)	6 (20.0)	66 (31.4)

None of the control isolates was positive for *cnf‐*1, the least prevalent gene among the investigated virulence genes. This finding is in accordance with a previous report where *cnf‐*1 was one of the less prevalent genes (Zhao et al., 2009). Between urine and fecal isolates from patients and healthy individuals, 5 (5.5%), 20 (22.2%), and 8 (26.6%) isolates were negative for all of the investigated virulence genes, respectively.

### 3.4 The relationship between virulence factors and phylogenetic groups

On the basis of previous studies, phylogenetic groups B2 and D usually possess virulence genes which enhance colonic persistence and adhesion in the urinary tract. These groups mainly consist of ExPEC, including UPEC (Micenková et al., 2016). In line with these studies, our results showed that most of the investigated virulence genes were more prevalent among urine isolates, but this was not statistically significant except for *fimH* and *ompT* (*p* < 0.05). Among the urine isolates, most of the virulence genes were concentrated within phylogenetic group B2, whereas among the fecal isolates of the patients and healthy individuals, they were concentrated in group D. Considering phylogenetic groups and virulence factors, there were 23 groups of urine isolates which were similar to the same host fecal isolates. Among them, 11 groups consisted of a pair of urine and fecal isolates, four groups had two urine and one fecal isolates, three groups had one urine and two fecal isolates, one group consisted of all the obtained urine and fecal isolates of one patient, one group had three urine and one fecal isolates, and the last three groups had one urine and three fecal isolates.

### 3.5 Antibiotic susceptibility tests

In the present study, the antibiotic susceptibility of the isolates to commonly used antibiotics for UTI was determined in order to find the best antibiotic therapeutic pattern. The prevalence of antibiotic resistance among *E. coli* isolates is demonstrated in Table 4. The isolates obtained from urine samples, as observed previously, were more sensitive to imipenem, amikacin, and ciprofloxacin. In contrast to the World Health Organization (WHO) recommendation for UTIs treatment with trimethoprim‐sulfamethoxazole, the current study indicated that the urine isolates had the highest level of resistance to trimethoprim‐sulfamethoxazole (51.1%). This is in agreement with several other studies which have reported that this antibiotic is an unsuitable treatment for UTI (Abass, Ali, & Authman, 2014; Totsika et al., 2013). A high sensitivity of *E. coli* strains to imipenem was detected earlier (Guidoni et al., 2008). Farshad et al. reported no resistance to imipenem in UPEC isolates in Iran (Farshad, Japoni, & Hosseini, 2008). Since imipenem is an antimicrobial agent of last resort and amikacin is not recommended for mild‐to‐moderate infections, these antibiotics cannot be recommended as choice drugs for the treatment of UTI caused by UPEC, although urine isolates were highly sensitive to these antibiotics. So, it seems that ciprofloxacin can be used as a choice drug for this infection.

**Table 4:** The number (%) of resistant isolates to the investigated antibiotics

Antibiotic	Source of isolates			Total (%)
	Controls’ fecal (%)	Patients’ fecal (%)	Urine (%)	
Ceftazidime	5 (16.7)	27 (30.0)	43 (47.8)	75 (35.7)
Trimethoprim‐sulfamethoxazole	12 (40.0)	5 (61.1)	46 (51.1)	113 (53.8)
Cefixime	7 (23.3)	32 (35.6)	42 (46.7)	81 (38.6)
Gentamicin	13 (43.3)	35 (38.9)	41 (45.6)	89 (42.4)
Ciprofloxacin	3 (10.0)	31 (34.4)	29 (32.2)	63 (30.0)
Ceftriaxone	9 (30.0)	30 (33.3)	34 (37.8)	73 (34.8)
Cefotaxime	5 (16.7)	23 (25.6)	34 (37.8)	62 (29.5)
Cefalexin	14 (46.7)	41 (45.6)	43 (47.8)	84 (46.7)
Nalidixic acid	10 (33.3)	34 (37.8)	45 (50.0)	89 (42.4)
Amikacin	11 (36.7)	31 (34.4)	30 (33.3)	73 (34.3)
Nitrofurantoin	7 (23.3)	23 (25.6)	32 (35.6)	62 (29.5)
Imipenem	6 (20.0)	19 (21.1)	27 (30.0)	52 (24.8)

Compared with the control isolates, urine isolates were significantly more resistant to ceftazidime, cefotaxime, ciprofloxacin, and cefixime (*p* < 0.05). Compared to healthy control individuals, fecal isolates from patients were significantly more resistant to ciprofloxacin (*p* < 0.05). Urine isolates were more resistant to ceftazidime than fecal isolates of the patients. Moreover, the antibiotic resistance rates to most antibiotics were higher in urine isolates. The isolates of three groups had a high level of resistance to trimethoprim‐sulfamethoxazole, gentamicin, cephalexin, and nalidixic acid, which could be due to the illogical use rate of antibiotics and the transfer of plasmids between bacteria (Uma, Prabhakar, Rajendran, Kavitha, & Sarayu, 2009).

Susceptibility to all tested antibiotics was observed in 17.7%, 16.6%, and 6.6% of urine and fecal isolates from patients and healthy individuals, respectively. Seven (7.7%) and two isolates (2.2%) among urine and patients’ fecal isolates were resistant to all of the investigated antibiotics, respectively. Multidrug resistance was observed in 63.3% of urine isolates, 54.4% of fecal isolates of patients, and 56.6% of healthy individuals’ fecal isolates, which is a public health concern in several countries (Ulett et al., 2013).

### 3.6 Pulsed‐field gel electrophoresis

One hundred and thirty‐two PFGE profiles were obtained from the genome of 140 examined *E. coli* isolates. Fifty‐four (40.90%) profiles were found only in urine isolates, 57 (43.18%) profiles were found only in fecal isolates of the patients, 19 (14.39%) profiles were found only in fecal isolates of healthy women, and two (1.51%) profiles were shared between urine and fecal isolates of the same patient. Applying the criterion of 80% similarity of the PFGE profiles to the PFGE profiles of the isolates, it was found that the host's urine profiles were identified within the host's fecal microbiota of eight (26.6%) patients. In two of these patients, one of the fecal isolates was genetically indistinguishable from one of the host's urine isolates (100% PFGE profiles similarity). The fecal isolates of the control group were not related to the urine isolates. The most investigated patients (22 patients; 73.3%) had distinct urine profiles which were not similar to any of their fecal isolates. Previous studies, by contrast, have reported that fecal clones can be mostly isolated from urinary tract infection (Moreno et al., 2008; Srivastava et al., 2014). These differences can be explained in part by the number of isolates examined and the time of sampling. Schlager, Hendley, Bell, and Whittam (2002) showed that the dominant fecal strain changes from week to week. A previous study examined women with a history of UTI and indicated that the infecting strain was in the feces for such a short time before the development of UTI (Richards & Cooke, 1979), hence the fact that the urine clone might truly had been absent from the stool at the time of the study. These clones may migrate to the vaginal and periurethral reservoirs at the time of sampling (Moreno et al., 2006). It is also possible that UTI was caused by the nondominant fecal clones which were missing because of the limited sampling, or it might be that they were directly transmitted by sexual contact (Foxman et al., 2002).

The comparison of urine and fecal isolates from the same patient with similar PFGE profiles showed that genetically indistinguishable isolates (100% similarity) were identical based on the virulence genes profile and antibiotic susceptibility pattern. The other isolates from the same patient which showed PFGE profiles with more than 80% similarity had the same virulence genes profiles but were different considering the antibiotic susceptibility patterns.

In conclusion, based on these findings that most of the patients had fecal isolates with distinct PFGE profiles and virulence genes were more prevalent among urine isolates, the special pathogenicity hypothesis is supported. Despite this, according to the identification of genetically related fecal isolates to host's urine clones in 26.6% of the patients, certain roles to the prevalence theory are assigned.

## CONFLICT OF INTEREST

The authors declare that there is no conflict of interest.

## AUTHORS CONTRIBUTION

MB performed all the experiments and PFGE analysis. AD and MM designed and directed the project. RF helped in planning the project. AMB carried out results analysis and wrote the manuscript in consultation with AD and RF. MA contributed to sample collection. ZN was involved in PFGE procedure. All authors read, edited, and approved the final version of the manuscript.

## ETHICS STATEMENT

The study protocol was approved by Shiraz Medical University Ethics Committee, Shiraz, Iran and all participants were informed about the study and signed the consent forms.

## Data Availability

All data are provided in full in the results section of this paper.
